# *catena*-Poly[[[tetra­aqua­erbium(III)]-μ-oxalato-κ^4^ *O* ^1^,*O* ^2^:*O* ^1′^,*O* ^2′^] [bromidobis(pyrazine-2-carboxyl­ato-κ^2^ *N* ^1^,*O*)cuprate(II)] tetra­hydrate]

**DOI:** 10.1107/S1600536810025274

**Published:** 2010-07-03

**Authors:** Hui-Fu Yang, Shi-Hai Xu

**Affiliations:** a Department of Chemistry, Jinan University, Guangzhou 510632, People’s Republic of China

## Abstract

In the title heterometallic complex, {[Er(C_2_O_4_)(H_2_O)_4_][CuBr(C_5_H_3_N_2_O_2_)_2_]·4H_2_O}_*n*_, the Er^III^ atom is eight-coordin­ated by four O atoms from two centrosymmetric oxalate ligands and four water mol­ecules, displaying a bicapped trigonal-prismatic geometry. The oxalate ligands bridge the Er atoms into a polymeric cationic chain along [110]. The Cu^II^ atom is five-coordinated in a square-pyramidal geometry by two pyrazine-2-carboxyl­ate ligands and a Br atom, forming a discrete anion. The polymeric cations, complex anions and uncoordinated water mol­ecules are self-assembled into a three-dimensional supra­molecular network through O—H⋯N, O—H⋯O and O—H⋯Br hydrogen bonds.

## Related literature

For general background to the topologies and potential applications of transition metal–lanthanide complexes, see: Barbour (2006 ▶); Kong *et al.* (2008 ▶); Rao *et al.* (2004 ▶); Zhang *et al.* (2005 ▶); Zhao *et al.* (2003 ▶). For general background to transition metal–lanthanide complexes with organic ligands containing mixed-donor atoms, see: Costes *et al.* (2004 ▶); Deng *et al.* (1996 ▶); He *et al.* (2005 ▶); Liang *et al.* (2001 ▶); Mahata *et al.* (2005 ▶); Ma, Liu *et al.* (2009 ▶); Zhang *et al.* (2004 ▶). For heterometallic complexes constructed from pyrazine-2-carb­oxy­lic acid, see: Deng *et al.* (2008 ▶); Feng & Wen (2009 ▶). For general background to *in situ* reactions, see: Li *et al.* (2006 ▶); Ma, Zeng *et al.* (2009 ▶).
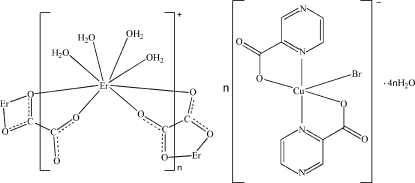


## Experimental

### 

#### Crystal data

[Er(C_2_O_4_)(H_2_O)_4_][CuBr(C_5_H_3_N_2_O_2_)_2_]·4H_2_O
*M* *_r_* = 789.05
Triclinic, 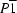

*a* = 8.6678 (3) Å
*b* = 10.2623 (4) Å
*c* = 13.8748 (2) Å
α = 96.872 (1)°
β = 99.419 (1)°
γ = 99.748 (1)°
*V* = 1186.10 (6) Å^3^
*Z* = 2
Mo *K*α radiation
μ = 6.18 mm^−1^
*T* = 295 K
0.26 × 0.25 × 0.19 mm

#### Data collection

Bruker APEXII CCD diffractometer
Absorption correction: multi-scan (*SADABS*; Sheldrick, 1996 ▶) *T*_min_ = 0.232, *T*_max_ = 0.324
14850 measured reflections
5260 independent reflections
4480 reflections with *I* > 2σ(*I*)
*R*_int_ = 0.023

#### Refinement

*R*[*F* ^2^ > 2σ(*F* ^2^)] = 0.027
*wR*(*F* ^2^) = 0.072
*S* = 1.01
5260 reflections
316 parameters
24 restraints
H-atom parameters constrained
Δρ_max_ = 1.59 e Å^−3^
Δρ_min_ = −0.88 e Å^−3^

### 

Data collection: *APEX2* (Bruker, 2007 ▶); cell refinement: *SAINT* (Bruker, 2007 ▶); data reduction: *SAINT*; program(s) used to solve structure: *SHELXS97* (Sheldrick, 2008 ▶); program(s) used to refine structure: *SHELXL97* (Sheldrick, 2008 ▶); molecular graphics: *SHELXTL* (Sheldrick, 2008 ▶); software used to prepare material for publication: *SHELXTL*.

## Supplementary Material

Crystal structure: contains datablocks I, global. DOI: 10.1107/S1600536810025274/hy2323sup1.cif

Structure factors: contains datablocks I. DOI: 10.1107/S1600536810025274/hy2323Isup2.hkl

Additional supplementary materials:  crystallographic information; 3D view; checkCIF report

## Figures and Tables

**Table 1:** Hydrogen-bond geometry (Å, °)

*D*—H⋯*A*	*D*—H	H⋯*A*	*D*⋯*A*	*D*—H⋯*A*
O1*W*—H1*W*⋯O5*W*^i^	0.82	1.92	2.712 (3)	163
O1*W*—H2*W*⋯N2^ii^	0.82	2.01	2.823 (4)	168
O2*W*—H3*W*⋯O4^iii^	0.82	2.04	2.825 (3)	159
O2*W*—H4*W*⋯O7*W*^iv^	0.82	1.97	2.788 (4)	174
O3*W*—H5*W*⋯O2^v^	0.82	1.97	2.781 (3)	167
O3*W*—H6*W*⋯O5*W*^vi^	0.82	1.89	2.710 (4)	176
O4*W*—H7*W*⋯O7*W*^vi^	0.82	1.94	2.758 (3)	178
O4*W*—H8*W*⋯N4	0.82	2.08	2.886 (4)	169
O5*W*—H9*W*⋯O8*W*	0.82	1.92	2.670 (4)	152
O5*W*—H10*W*⋯O6*W*^vii^	0.82	2.03	2.835 (4)	168
O6*W*—H11*W*⋯Br1^viii^	0.82	2.59	3.299 (3)	146
O6*W*—H12*W*⋯O3^ix^	0.82	2.57	3.238 (4)	139
O6*W*—H12*W*⋯O4^ix^	0.82	2.07	2.866 (4)	164
O7*W*—H13*W*⋯O5	0.82	2.04	2.826 (3)	161
O7*W*—H14*W*⋯O6*W*	0.82	1.94	2.746 (4)	167
O8*W*—H15*W*⋯O1^viii^	0.82	2.58	3.351 (4)	157
O8*W*—H15*W*⋯O2^viii^	0.82	2.23	2.951 (4)	147
O8*W*—H16*W*⋯Br1^vi^	0.82	2.53	3.337 (3)	170

Symmetry codes: (i) 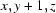; (ii) 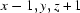; (iii) 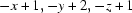; (iv) 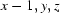; (v) 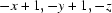; (vi) 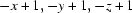; (vii) 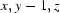; (viii) 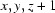; (ix) 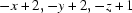.

**Fig. 1.**
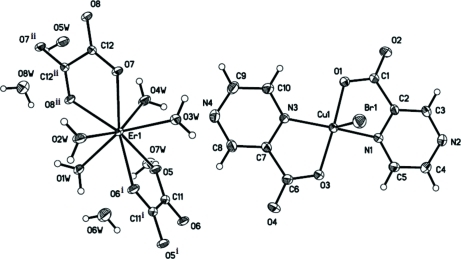
The structure of the title compound, showing the 30% probability displacement ellipsoids. H atoms have been omitted for clarity. [Symmetry codes: (i) 2-x, -y, 1-z; (ii) 1-x, -y, 1-z.]

**Fig. 2.**
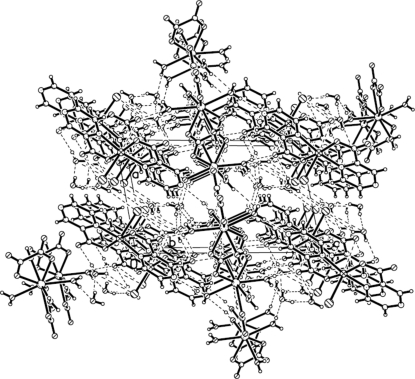
A packing view of the title compound, showing the intermolecular hydrogen bonds (dashed lines).

## supplementary crystallographic information

### Comment

The design and construction of transition–lanthanide metal complexes has gained great recognition over the last decade because of their intriguing network topolopies and potential applications, and due to their magnetic properties, their capacity for gas storage, as luminescent materials, and so on (Barbour, 2006; Kong *et al.*, 2008; Rao *et al.*, 2004; Zhang *et al.*, 2005; Zhao *et al.*, 2003). So far, numerous heterometallic complexes have been obtained by allowing the assembly of mixed metal ions and organic ligands containing mixed-donor atoms similar to the established methods used in traditional transition metal chemistry, such as pyridinecarboxylate, pyrazinecarboxylate, carbonyl, CN group, amino acids and so on (Costes *et al.*, 2004; Deng *et al.*, 1996; He *et al.*, 2005; Liang *et al.*, 2001; Mahata *et al.*, 2005; Ma, Liu *et al.*, 2009; Zhang *et al.*, 2004). Pyrazine-2-carboxylic acid (2-Hpzc) is a multifunctional bridging ligand possessing of O and N donors, which can thus be chosen to construct heterometallic complexes, as is reported in literature (Deng *et al.*, 2008; Feng & Wen, 2009). In this paper, we describe the synthesis and structure of a heterometallic hybrid compound obtained by the reaction of 2-Hpzc with Er_2_O_3_ and CuBr_2_*via* a hydrothermal method. Since no oxalate was directly introduced into the starting reaction mixture, we suppose that the oxalate ligand was synthesized *in situ* reactions.

Firstly, the decarboxylation reaction of the 2-Hpzc occured under high temperatures, forming CO_2_. Then, the oxalate anion was formed *via* the *in situ* reductive coupling of CO_2_. Actually, such *in situ* reactions have been reported in literature [Li *et al.*, 2006; Ma, Zeng *et al.*, 2009]. As depicted in Fig. 1, the title compound is composed of an [Er(C_2_O_4_)(H_2_O)_4_]^+^ cation, a [Cu(2-pzc)_2_Br]^-^ anion and four uncoordinated water molecules. The Er^III^ atom lies in the region of *z* close to 0.5 and the Cu^II^ atom in the region of *z* close to 0. The Er^III^ center is eight-coordinated by four carboxylate O atoms from two oxalate ligands and four water molecules, displaying a bicapped trigonal-prismatic geometry. The coordination geometry around the Cu^II^ center can be described as square-pyramidal, defined by two O and two N atoms from two 2-pzc ligands and one Br atom. The oxalate ligands bridge the Er atoms into a polymeric cationic chain along [1 1 0], with Er···Er separations of 6.176 (2) and 6.088 (3) Å. The Cu atoms form a simple dimer by a Cu···O contact [3.127 (2) Å], with a Cu···Cu separation of 5.321 (2) Å. The linear coordination polymers, discrete dimers and uncoordinated water molecules are further self-assembled into a three-dimensional supramolecular network structure *via* intermolecular O—H···O, O—H···N and O—H···Br hydrogen bonds involving the carboxylate O atoms of the 2-pzc ligands, the O atoms from coordinated and uncoordinated water molecules and Br atom (Fig. 2, Table 1).

### Experimental

A mixture of copper bromide (0.5 mmol, 0.112 g), 2-Hpzc (0.5 mmol, 0.062 g), erbium oxide (0.52 mmol, 0.096 g), HNO_3_ (1 ml) and H_2_O (10 ml) was stirred for 30 min in air and then sealed in a 23 ml Teflon-lined reactor and kept under autogenous pressure at 423 K for 72 h. The mixture was cooled to room temperature at a rate of 10 K h^-1^. The purple block crystals were obtained in a yield of 42% based on Er.

### Refinement

C-bound H atoms were placed at calculated positions and were treated as riding on the parent C atoms, with C—H = 0.93 Å and with *U*_iso_(H) = 1.2*U*_eq_(C). Water H atoms were tentatively located in difference Fourier maps and were refined as riding, with distance restraints of O—H = 0.82 and H···H = 1.32 Å and with *U*_iso_(H) = 1.5*U*_eq_(O). The highest residual electron density was found 1.59 Å from atom Cu1 and the deepest hole 0.88 Å from atom Er1.

### Crystal data

**Table:**

[Er(C_2_O_4_)(H_2_O)_4_][CuBr(C_5_H_3_N_2_O_2_)_2_]·4H_2_O	*Z* = 2
*M**_r_* = 789.05	*F*(000) = 764
Triclinic, *P*1	*D*_x_ = 2.209 Mg m^−^^3^
Hall symbol: -P 1	Mo *K*α radiation, λ = 0.71073 Å
*a* = 8.6678 (3) Å	Cell parameters from 5837 reflections
*b* = 10.2623 (4) Å	θ = 2.8–27.9°
*c* = 13.8748 (2) Å	µ = 6.18 mm^−^^1^
α = 96.872 (1)°	*T* = 295 K
β = 99.419 (1)°	Block, purple
γ = 99.748 (1)°	0.26 × 0.25 × 0.19 mm
*V* = 1186.10 (6) Å^3^	

### Data collection

**Table:**

Bruker APEXII CCD diffractometer	5260 independent reflections
Radiation source: fine-focus sealed tube	4480 reflections with *I* > 2σ(*I*)
graphite	*R*_int_ = 0.023
φ and ω scan	θ_max_ = 27.5°, θ_min_ = 1.5°
Absorption correction: multi-scan (*SADABS*; Sheldrick, 1996)	*h* = −10→11
*T*_min_ = 0.232, *T*_max_ = 0.324	*k* = −13→13
14850 measured reflections	*l* = −18→17

### Refinement

**Table:**

Refinement on *F*^2^	Primary atom site location: structure-invariant direct methods
Least-squares matrix: full	Secondary atom site location: difference Fourier map
*R*[*F*^2^ > 2σ(*F*^2^)] = 0.027	Hydrogen site location: inferred from neighbouring sites
*wR*(*F*^2^) = 0.072	H-atom parameters constrained
*S* = 1.01	*w* = 1/[σ^2^(*F*_o_^2^) + (0.0399*P*)^2^ + 0.5356*P*] where *P* = (*F*_o_^2^ + 2*F*_c_^2^)/3
5260 reflections	(Δ/σ)_max_ = 0.001
316 parameters	Δρ_max_ = 1.59 e Å^−^^3^
24 restraints	Δρ_min_ = −0.88 e Å^−^^3^

### Fractional atomic coordinates and isotropic or equivalent isotropic displacement parameters (Å^2^)

**Table:**

	*x*	*y*	*z*	*U*_iso_*/*U*_eq_	
Er1	0.242464 (16)	0.750762 (12)	0.496898 (10)	0.02163 (6)	
Br1	0.63182 (5)	0.80237 (4)	−0.05645 (3)	0.04494 (11)	
Cu1	0.86112 (6)	0.70254 (4)	0.05289 (3)	0.03103 (11)	
O1	0.8075 (3)	0.5245 (2)	−0.02710 (18)	0.0358 (6)	
O2	0.8561 (4)	0.4105 (3)	−0.1609 (2)	0.0546 (8)	
O3	0.9505 (3)	0.8638 (2)	0.14745 (18)	0.0371 (6)	
O4	0.9256 (4)	0.9694 (3)	0.29159 (19)	0.0465 (7)	
O5	0.5154 (3)	0.8333 (2)	0.49529 (17)	0.0275 (5)	
O6	0.7024 (3)	1.0134 (2)	0.49841 (18)	0.0288 (5)	
O7	0.0196 (3)	0.5961 (2)	0.40579 (16)	0.0305 (6)	
O8	−0.1564 (3)	0.4115 (2)	0.40878 (17)	0.0310 (6)	
N1	1.0142 (3)	0.7379 (3)	−0.03731 (19)	0.0270 (6)	
N2	1.1892 (4)	0.7526 (3)	−0.1867 (2)	0.0360 (7)	
N3	0.7441 (4)	0.6513 (3)	0.1585 (2)	0.0289 (6)	
N4	0.5947 (4)	0.6246 (3)	0.3185 (2)	0.0413 (8)	
C1	0.8766 (5)	0.5131 (3)	−0.1011 (2)	0.0320 (8)	
C2	0.9942 (4)	0.6345 (3)	−0.1109 (2)	0.0271 (7)	
C3	1.0800 (4)	0.6438 (4)	−0.1856 (3)	0.0326 (8)	
H3	1.0617	0.5725	−0.2369	0.039*	
C4	1.2098 (5)	0.8521 (4)	−0.1124 (3)	0.0358 (9)	
H4	1.2867	0.9281	−0.1101	0.043*	
C5	1.1210 (4)	0.8469 (3)	−0.0382 (3)	0.0317 (8)	
H5	1.1361	0.9199	0.0113	0.038*	
C6	0.8932 (4)	0.8711 (3)	0.2268 (3)	0.0310 (8)	
C7	0.7757 (4)	0.7492 (3)	0.2364 (2)	0.0282 (7)	
C8	0.6999 (5)	0.7350 (4)	0.3157 (3)	0.0353 (8)	
H8	0.7226	0.8043	0.3687	0.042*	
C9	0.5669 (5)	0.5284 (4)	0.2412 (3)	0.0433 (10)	
H9	0.4962	0.4495	0.2417	0.052*	
C10	0.6391 (5)	0.5408 (4)	0.1601 (3)	0.0384 (9)	
H10	0.6144	0.4720	0.1067	0.046*	
C11	0.5638 (4)	0.9558 (3)	0.4983 (2)	0.0228 (7)	
C12	−0.0399 (4)	0.5024 (3)	0.4461 (2)	0.0241 (7)	
O1W	0.3563 (3)	0.8151 (3)	0.66122 (17)	0.0430 (7)	
H1W	0.4129	0.8882	0.6845	0.064*	
H2W	0.3144	0.7877	0.7057	0.064*	
O2W	0.0271 (3)	0.8392 (2)	0.5368 (2)	0.0409 (7)	
H3W	0.0474	0.9082	0.5768	0.061*	
H4W	−0.0580	0.7965	0.5443	0.061*	
O3W	0.2166 (3)	0.7760 (2)	0.33238 (17)	0.0375 (6)	
H5W	0.1866	0.7138	0.2865	0.056*	
H6W	0.2701	0.8366	0.3118	0.056*	
O4W	0.3673 (3)	0.5775 (2)	0.4469 (2)	0.0384 (6)	
H7W	0.3347	0.4964	0.4398	0.058*	
H8W	0.4410	0.5907	0.4168	0.058*	
O5W	0.5945 (3)	0.0292 (3)	0.7338 (2)	0.0478 (7)	
H9W	0.5749	0.0653	0.7851	0.072*	
H10W	0.6603	−0.0171	0.7494	0.072*	
O6W	0.8142 (4)	0.8544 (3)	0.7569 (2)	0.0604 (9)	
H12W	0.8929	0.9105	0.7552	0.091*	
H11W	0.8110	0.8507	0.8152	0.091*	
O7W	0.7480 (3)	0.6941 (2)	0.5762 (2)	0.0387 (6)	
H13W	0.6919	0.7345	0.5421	0.058*	
H14W	0.7622	0.7313	0.6336	0.058*	
O8W	0.5890 (4)	0.2199 (3)	0.8812 (2)	0.0631 (9)	
H15W	0.6508	0.2917	0.8877	0.095*	
H16W	0.5431	0.2229	0.9284	0.095*	

### Atomic displacement parameters (Å^2^)

**Table:**

	*U*^11^	*U*^22^	*U*^33^	*U*^12^	*U*^13^	*U*^23^
Er1	0.02294 (10)	0.01592 (8)	0.02381 (9)	−0.00464 (6)	0.00914 (6)	−0.00033 (6)
Br1	0.0424 (2)	0.0477 (2)	0.0447 (2)	0.00720 (19)	0.00907 (19)	0.00745 (19)
Cu1	0.0406 (3)	0.0249 (2)	0.0266 (2)	−0.00133 (19)	0.0166 (2)	−0.00292 (17)
O1	0.0452 (16)	0.0277 (12)	0.0329 (13)	−0.0031 (11)	0.0188 (12)	−0.0028 (10)
O2	0.081 (2)	0.0332 (14)	0.0424 (16)	−0.0094 (15)	0.0276 (16)	−0.0142 (12)
O3	0.0457 (16)	0.0303 (13)	0.0318 (14)	−0.0056 (12)	0.0169 (12)	−0.0033 (11)
O4	0.0581 (19)	0.0359 (14)	0.0379 (15)	−0.0042 (13)	0.0151 (14)	−0.0137 (12)
O5	0.0262 (12)	0.0159 (10)	0.0394 (14)	−0.0024 (9)	0.0106 (11)	0.0025 (10)
O6	0.0252 (13)	0.0185 (10)	0.0421 (14)	−0.0032 (10)	0.0138 (11)	0.0019 (10)
O7	0.0358 (14)	0.0237 (11)	0.0257 (12)	−0.0112 (10)	0.0053 (11)	0.0037 (10)
O8	0.0319 (13)	0.0282 (12)	0.0262 (12)	−0.0127 (10)	0.0053 (11)	0.0038 (10)
N1	0.0311 (16)	0.0243 (14)	0.0237 (14)	0.0014 (12)	0.0066 (12)	−0.0002 (11)
N2	0.0399 (18)	0.0391 (17)	0.0304 (16)	0.0028 (15)	0.0147 (14)	0.0063 (13)
N3	0.0342 (16)	0.0237 (14)	0.0293 (15)	0.0023 (12)	0.0133 (13)	−0.0002 (12)
N4	0.048 (2)	0.0419 (18)	0.0375 (18)	0.0063 (16)	0.0211 (16)	0.0071 (15)
C1	0.044 (2)	0.0243 (16)	0.0255 (17)	0.0000 (16)	0.0110 (16)	−0.0007 (14)
C2	0.0344 (19)	0.0274 (16)	0.0204 (16)	0.0061 (15)	0.0082 (14)	0.0024 (13)
C3	0.039 (2)	0.0337 (18)	0.0246 (17)	0.0051 (17)	0.0110 (16)	−0.0001 (15)
C4	0.035 (2)	0.0352 (19)	0.036 (2)	−0.0019 (17)	0.0102 (17)	0.0057 (16)
C5	0.0326 (19)	0.0286 (17)	0.0312 (19)	0.0014 (15)	0.0071 (16)	−0.0004 (14)
C6	0.0318 (19)	0.0271 (17)	0.0326 (19)	0.0021 (15)	0.0093 (16)	−0.0005 (15)
C7	0.0325 (19)	0.0268 (17)	0.0258 (17)	0.0069 (15)	0.0082 (15)	0.0009 (14)
C8	0.045 (2)	0.0345 (19)	0.0285 (19)	0.0096 (17)	0.0132 (17)	0.0025 (15)
C9	0.047 (2)	0.034 (2)	0.052 (2)	0.0019 (18)	0.025 (2)	0.0071 (18)
C10	0.044 (2)	0.0285 (18)	0.041 (2)	0.0000 (17)	0.0159 (18)	−0.0013 (16)
C11	0.0250 (17)	0.0201 (15)	0.0221 (16)	−0.0002 (14)	0.0081 (14)	−0.0002 (12)
C12	0.0269 (18)	0.0205 (15)	0.0227 (17)	−0.0021 (14)	0.0106 (14)	−0.0033 (13)
O1W	0.0475 (17)	0.0427 (15)	0.0261 (13)	−0.0250 (13)	0.0108 (12)	−0.0022 (11)
O2W	0.0306 (14)	0.0292 (13)	0.0579 (17)	−0.0072 (11)	0.0199 (13)	−0.0102 (12)
O3W	0.0540 (17)	0.0287 (12)	0.0239 (12)	−0.0092 (12)	0.0094 (12)	0.0016 (10)
O4W	0.0421 (15)	0.0188 (11)	0.0575 (17)	0.0002 (11)	0.0276 (13)	0.0015 (11)
O5W	0.0489 (18)	0.0379 (15)	0.0496 (17)	−0.0084 (13)	0.0054 (14)	0.0076 (13)
O6W	0.060 (2)	0.061 (2)	0.0511 (19)	−0.0132 (17)	0.0087 (16)	0.0095 (16)
O7W	0.0362 (15)	0.0275 (12)	0.0513 (16)	0.0036 (11)	0.0088 (13)	0.0043 (12)
O8W	0.061 (2)	0.059 (2)	0.067 (2)	−0.0046 (17)	0.0265 (18)	0.0014 (17)

### Geometric parameters (Å, °)

**Table:**

Er1—O1W	2.300 (2)	C1—C2	1.504 (5)
Er1—O3W	2.307 (2)	C2—C3	1.375 (5)
Er1—O2W	2.326 (2)	C3—H3	0.9300
Er1—O4W	2.327 (2)	C4—C5	1.384 (5)
Er1—O8^i^	2.337 (2)	C4—H4	0.9300
Er1—O7	2.352 (2)	C5—H5	0.9300
Er1—O6^ii^	2.377 (2)	C6—C7	1.508 (5)
Er1—O5	2.379 (2)	C7—C8	1.382 (5)
Br1—Cu1	2.7158 (7)	C8—H8	0.9300
Cu1—O3	1.943 (2)	C9—C10	1.383 (5)
Cu1—O1	1.961 (2)	C9—H9	0.9300
Cu1—N3	1.985 (3)	C10—H10	0.9300
Cu1—N1	1.987 (3)	C11—C11^ii^	1.547 (6)
O1—C1	1.274 (4)	C12—C12^i^	1.553 (6)
O2—C1	1.228 (4)	O1W—H1W	0.8200
O3—C6	1.278 (4)	O1W—H2W	0.8200
O4—C6	1.229 (4)	O2W—H3W	0.8200
O5—C11	1.250 (4)	O2W—H4W	0.8200
O6—C11	1.245 (4)	O3W—H5W	0.8200
O6—Er1^ii^	2.377 (2)	O3W—H6W	0.8200
O7—C12	1.248 (4)	O4W—H7W	0.8200
O8—C12	1.246 (4)	O4W—H8W	0.8200
O8—Er1^i^	2.337 (2)	O5W—H9W	0.8200
N1—C5	1.328 (4)	O5W—H10W	0.8200
N1—C2	1.349 (4)	O6W—H12W	0.8200
N2—C4	1.329 (5)	O6W—H11W	0.8200
N2—C3	1.340 (5)	O7W—H13W	0.8200
N3—C10	1.334 (5)	O7W—H14W	0.8200
N3—C7	1.342 (4)	O8W—H15W	0.8200
N4—C9	1.328 (5)	O8W—H16W	0.8200
N4—C8	1.337 (5)		
			
O1W—Er1—O3W	152.60 (9)	O2—C1—O1	124.3 (3)
O1W—Er1—O2W	85.77 (10)	O2—C1—C2	119.9 (3)
O3W—Er1—O2W	100.11 (10)	O1—C1—C2	115.8 (3)
O1W—Er1—O4W	103.65 (10)	N1—C2—C3	120.4 (3)
O3W—Er1—O4W	82.75 (9)	N1—C2—C1	114.4 (3)
O2W—Er1—O4W	153.97 (9)	C3—C2—C1	125.1 (3)
O1W—Er1—O8^i^	68.99 (8)	N2—C3—C2	121.9 (3)
O3W—Er1—O8^i^	138.02 (8)	N2—C3—H3	119.1
O2W—Er1—O8^i^	83.33 (9)	C2—C3—H3	119.1
O4W—Er1—O8^i^	77.78 (9)	N2—C4—C5	122.4 (3)
O1W—Er1—O7	136.09 (9)	N2—C4—H4	118.8
O3W—Er1—O7	70.96 (8)	C5—C4—H4	118.8
O2W—Er1—O7	76.34 (8)	N1—C5—C4	120.3 (3)
O4W—Er1—O7	80.30 (9)	N1—C5—H5	119.9
O8^i^—Er1—O7	69.32 (8)	C4—C5—H5	119.9
O1W—Er1—O6^ii^	80.45 (9)	O4—C6—O3	124.5 (3)
O3W—Er1—O6^ii^	76.48 (8)	O4—C6—C7	120.2 (3)
O2W—Er1—O6^ii^	70.57 (8)	O3—C6—C7	115.3 (3)
O4W—Er1—O6^ii^	134.44 (9)	N3—C7—C8	120.3 (3)
O8^i^—Er1—O6^ii^	141.07 (8)	N3—C7—C6	114.6 (3)
O7—Er1—O6^ii^	127.85 (8)	C8—C7—C6	125.1 (3)
O1W—Er1—O5	75.95 (9)	N4—C8—C7	121.9 (3)
O3W—Er1—O5	81.80 (9)	N4—C8—H8	119.1
O2W—Er1—O5	136.79 (8)	C7—C8—H8	119.1
O4W—Er1—O5	69.22 (8)	N4—C9—C10	122.5 (4)
O8^i^—Er1—O5	123.61 (8)	N4—C9—H9	118.7
O7—Er1—O5	141.32 (8)	C10—C9—H9	118.7
O6^ii^—Er1—O5	67.99 (7)	N3—C10—C9	120.0 (3)
O3—Cu1—O1	167.79 (12)	N3—C10—H10	120.0
O3—Cu1—N3	83.39 (11)	C9—C10—H10	120.0
O1—Cu1—N3	95.48 (11)	O6—C11—O5	127.1 (3)
O3—Cu1—N1	95.47 (11)	O6—C11—C11^ii^	117.2 (3)
O1—Cu1—N1	83.09 (10)	O5—C11—C11^ii^	115.8 (4)
N3—Cu1—N1	167.97 (12)	O7—C12—O8	127.0 (3)
O3—Cu1—Br1	97.11 (8)	O7—C12—C12^i^	116.8 (3)
O1—Cu1—Br1	95.08 (8)	O8—C12—C12^i^	116.3 (4)
N3—Cu1—Br1	98.49 (9)	Er1—O1W—H1W	124.5
N1—Cu1—Br1	93.53 (8)	Er1—O1W—H2W	122.6
C1—O1—Cu1	114.6 (2)	H1W—O1W—H2W	106.9
C6—O3—Cu1	115.0 (2)	Er1—O2W—H3W	116.9
C11—O5—Er1	119.8 (2)	Er1—O2W—H4W	126.3
C11—O6—Er1^ii^	119.3 (2)	H3W—O2W—H4W	107.1
C12—O7—Er1	118.4 (2)	Er1—O3W—H5W	124.0
C12—O8—Er1^i^	119.3 (2)	Er1—O3W—H6W	123.9
C5—N1—C2	118.2 (3)	H5W—O3W—H6W	107.0
C5—N1—Cu1	130.1 (2)	Er1—O4W—H7W	129.1
C2—N1—Cu1	111.5 (2)	Er1—O4W—H8W	120.7
C4—N2—C3	116.7 (3)	H7W—O4W—H8W	107.7
C10—N3—C7	118.4 (3)	H9W—O5W—H10W	107.1
C10—N3—Cu1	129.9 (2)	H12W—O6W—H11W	107.2
C7—N3—Cu1	111.6 (2)	H13W—O7W—H14W	107.4
C9—N4—C8	116.8 (3)	H15W—O8W—H16W	106.9
			
O3—Cu1—O1—C1	−89.6 (6)	C5—N1—C2—C3	−1.5 (5)
N3—Cu1—O1—C1	−173.7 (3)	Cu1—N1—C2—C3	174.7 (3)
N1—Cu1—O1—C1	−5.7 (3)	C5—N1—C2—C1	177.2 (3)
Br1—Cu1—O1—C1	87.2 (3)	Cu1—N1—C2—C1	−6.6 (4)
O1—Cu1—O3—C6	−89.5 (6)	O2—C1—C2—N1	−175.8 (3)
N3—Cu1—O3—C6	−4.1 (3)	O1—C1—C2—N1	2.1 (5)
N1—Cu1—O3—C6	−172.1 (3)	O2—C1—C2—C3	2.8 (6)
Br1—Cu1—O3—C6	93.7 (2)	O1—C1—C2—C3	−179.3 (3)
O1W—Er1—O5—C11	−84.7 (2)	C4—N2—C3—C2	−0.6 (5)
O3W—Er1—O5—C11	79.2 (2)	N1—C2—C3—N2	2.2 (5)
O2W—Er1—O5—C11	−16.8 (3)	C1—C2—C3—N2	−176.3 (3)
O4W—Er1—O5—C11	164.5 (3)	C3—N2—C4—C5	−1.7 (5)
O8^i^—Er1—O5—C11	−137.1 (2)	C2—N1—C5—C4	−0.7 (5)
O7—Er1—O5—C11	124.2 (2)	Cu1—N1—C5—C4	−176.1 (3)
O6^ii^—Er1—O5—C11	0.6 (2)	N2—C4—C5—N1	2.4 (6)
O1W—Er1—O7—C12	−19.6 (3)	Cu1—O3—C6—O4	−174.1 (3)
O3W—Er1—O7—C12	165.7 (3)	Cu1—O3—C6—C7	4.4 (4)
O2W—Er1—O7—C12	−88.3 (2)	C10—N3—C7—C8	−0.4 (5)
O4W—Er1—O7—C12	80.1 (2)	Cu1—N3—C7—C8	177.0 (3)
O8^i^—Er1—O7—C12	−0.4 (2)	C10—N3—C7—C6	−178.8 (3)
O6^ii^—Er1—O7—C12	−139.9 (2)	Cu1—N3—C7—C6	−1.4 (4)
O5—Er1—O7—C12	118.0 (2)	O4—C6—C7—N3	176.7 (3)
O3—Cu1—N1—C5	−9.8 (3)	O3—C6—C7—N3	−1.9 (5)
O1—Cu1—N1—C5	−177.6 (3)	O4—C6—C7—C8	−1.7 (6)
N3—Cu1—N1—C5	−93.8 (6)	O3—C6—C7—C8	179.8 (3)
Br1—Cu1—N1—C5	87.7 (3)	C9—N4—C8—C7	0.3 (6)
O3—Cu1—N1—C2	174.5 (2)	N3—C7—C8—N4	0.6 (6)
O1—Cu1—N1—C2	6.7 (2)	C6—C7—C8—N4	178.9 (3)
N3—Cu1—N1—C2	90.5 (6)	C8—N4—C9—C10	−1.4 (6)
Br1—Cu1—N1—C2	−88.0 (2)	C7—N3—C10—C9	−0.7 (5)
O3—Cu1—N3—C10	179.9 (3)	Cu1—N3—C10—C9	−177.5 (3)
O1—Cu1—N3—C10	−12.3 (3)	N4—C9—C10—N3	1.7 (6)
N1—Cu1—N3—C10	−94.9 (6)	Er1^ii^—O6—C11—O5	179.5 (3)
Br1—Cu1—N3—C10	83.7 (3)	Er1^ii^—O6—C11—C11^ii^	−0.3 (5)
O3—Cu1—N3—C7	2.9 (2)	Er1—O5—C11—O6	179.6 (3)
O1—Cu1—N3—C7	170.6 (2)	Er1—O5—C11—C11^ii^	−0.6 (4)
N1—Cu1—N3—C7	88.1 (6)	Er1—O7—C12—O8	−179.4 (3)
Br1—Cu1—N3—C7	−93.4 (2)	Er1—O7—C12—C12^i^	0.2 (5)
Cu1—O1—C1—O2	−178.5 (3)	Er1^i^—O8—C12—O7	−179.6 (3)
Cu1—O1—C1—C2	3.6 (4)	Er1^i^—O8—C12—C12^i^	0.8 (5)

Symmetry codes: (i) −*x*, −*y*+1, −*z*+1; (ii) −*x*+1, −*y*+2, −*z*+1.

### Hydrogen-bond geometry (Å, °)

**Table:**

*D*—H···*A*	*D*—H	H···*A*	*D*···*A*	*D*—H···*A*
O1W—H1W···O5W^iii^	0.82	1.92	2.712 (3)	163
O1W—H2W···N2^iv^	0.82	2.01	2.823 (4)	168
O2W—H3W···O4^ii^	0.82	2.04	2.825 (3)	159
O2W—H4W···O7W^v^	0.82	1.97	2.788 (4)	174
O3W—H5W···O2^vi^	0.82	1.97	2.781 (3)	167
O3W—H6W···O5W^vii^	0.82	1.89	2.710 (4)	176
O4W—H7W···O7W^vii^	0.82	1.94	2.758 (3)	178
O4W—H8W···N4	0.82	2.08	2.886 (4)	169
O5W—H9W···O8W	0.82	1.92	2.670 (4)	152
O5W—H10W···O6W^viii^	0.82	2.03	2.835 (4)	168
O6W—H11W···Br1^ix^	0.82	2.59	3.299 (3)	146
O6W—H12W···O3^x^	0.82	2.57	3.238 (4)	139
O6W—H12W···O4^x^	0.82	2.07	2.866 (4)	164
O7W—H13W···O5	0.82	2.04	2.826 (3)	161
O7W—H14W···O6W	0.82	1.94	2.746 (4)	167
O8W—H15W···O1^ix^	0.82	2.58	3.351 (4)	157
O8W—H15W···O2^ix^	0.82	2.23	2.951 (4)	147
O8W—H16W···Br1^vii^	0.82	2.53	3.337 (3)	170

Symmetry codes: (iii) *x*, *y*+1, *z*; (iv) *x*−1, *y*, *z*+1; (ii) −*x*+1, −*y*+2, −*z*+1; (v) *x*−1, *y*, *z*; (vi) −*x*+1, −*y*+1, −*z*; (vii) −*x*+1, −*y*+1, −*z*+1; (viii) *x*, *y*−1, *z*; (ix) *x*, *y*, *z*+1; (x) −*x*+2, −*y*+2, −*z*+1.
